# A new species of *Odorrana* (Anura, Ranidae) from Hunan Province, China

**DOI:** 10.3897/zookeys.1024.56399

**Published:** 2021-03-15

**Authors:** Bing Zhang, Yuan Li, Ke Hu, Pipeng Li, Zhirong Gu, Nengwen Xiao, Daode Yang

**Affiliations:** 1 Institute of Wildlife Conservation, Central South University of Forestry and Technology, Changsha 410004, China Central South University of Forestry and Technology Changsha China; 2 Institute of Herpetology, Shenyang Normal University, Shenyang 110034, China Shenyang Normal University Shenyang China; 3 Bureau of Hunan Badagongshan National Nature Reserve, Sangzhi 427100, China Bureau of Hunan Badagongshan National Nature Reserve Sangzhi China; 4 State Environmental Protection Key Laboratory of Regional Eco-process and Function Assessment, Chinese Research Academy of Environmental Sciences, Beijing 100012, China Chinese Research Academy of Environmental Sciences Beijing China

**Keywords:** Molecular phylogeny, morphology, *Odorrana sangzhiensis* sp. nov., phylogenetic analyses, taxonomy, Wuling Mountains

## Abstract

A new species, *Odorrana sangzhiensis***sp. nov.**, is described, based on five specimens from Sangzhi County, Zhangjiajie City, Hunan Province, China. Molecular phylogenetic analyses, based on mitochondrial 12S rRNA and 16S rRNA gene sequences, strongly support the new species as a monophyletic group nested into the *O. schmackeri* species complex. The new species can be distinguished from its congeners by a combination of the following characters: (1) body size medium (SVL: 42.1–45.1 mm in males, 83.3–92.7 mm in females); (2) dorsolateral folds absent; (3) tympanum diameter 1.53 times as long as the width of the disc of finger III in females; 2.3 times in males; (4) dorsal skin green with dense granules and sparse irregular brown spots; males with several large warts on dorsum; (5) two metacarpal tubercles; (6) relative finger lengths: I ≤ II < IV < III; (7) tibiotarsal articulation beyond the tip of the snout; (8) ventral surface smooth in females; throat and chest having pale spinules in adult males; (9) dorsal limbs green or yellow green with brown transverse bands; and (10) paired external vocal sacs located at corners of the throat, finger I with light yellow nuptial pad in males. This discovery increases the number of *Odorrana* species to 59 and those known from China to 37.

## Introduction

The odorous frogs of the genus *Odorrana* Fei, Ye & Huang, 1990 are distributed in tropical and subtropical Asia, including southern mainland China, Japan and Indochina west to north-eastern India, Myanmar and Thailand and south through Malaya and Sumatra to Borneo. There are 58 recognised species (Frost 2020). Most of the species inhabit mountain streams at elevations of 200–2000 m and can also be found on rocks or branches near the riverbed (Fei et al. 2009a; Frost 2020). Seven species from the genus *Odorrana* have recently been described using both molecular and morphological analyses (Chen et al. 2010a, b; Kuramoto et al. 2011; Mo et al. 2015; Wang et al. 2015; Pham et al. 2016; Li et al. 2018a). The phylogeny and diversity of *Odorrana* and the systematic status of taxa within the genus have been debated by taxonomists (Frost et al. 2006; Che et al. 2007; Fei et al. 2009a; Kurabayashi et al. 2010; Chen et al. 2013; Li et al. 2015). For example, the genus *Odorrana* was first recognised by Fei et al. (1990), while Dubois (1992) treated *Odorrana* as a subgenus of *Rana* and erected a new subgenus R. (Eburana). Frost et al. (2006) expanded the genus *Huia* to include both *Odorrana* and R. (Eburana). However, the monophyly of *Odorrana* was supported by subsequent analyses of mtDNA data, nuclear data and combinations of mtDNA and nuclear data and R. (Eburana) and *Huia* were rejected (Matsui et al. 2005; Stuart 2008; Pyron and Wiens 2011). Chen et al. (2013) used two mitochondrial genes to study the molecular phylogeny and diversity of *Odorrana* and they identified seven major clades.

*Odorrana schmackeri* Boettger, 1892 is a widely distributed species in subtropical and tropical regions in the south of the Qinling Mountains in China. Paleogeological events and climatic oscillation may have led to lineage divergence within *O. schmackeri* (Li et al. 2015). A comprehensive comparison of the morphological and molecular biological characteristics of *O. schmackeri* has revealed several cryptic species, such as *O. nanjiangensis* Fei, Ye, Xie & Jiang, 2007, *O. huanggangensis* Chen, Zhou & Zheng, 2010a, *O. tianmuii* Chen, Zhou & Zheng, 2010b and *O. kweichowensis* Li, Xu, Lv, Jiang, Wei & Wang, 2018. The phylogenetic relationships identified within the genus *Odorrana* has led to a proposed “*Odorrana schmackeri* species complex” (Li et al. 2015; Li et al. 2018a). The “*O. schmackeri* species complex” is characterised by species morphologically similar to *O. schmackeri* and contains several described species and several cryptic species: *O. hejiangensis*, *O. huanggangensis*, *O. kweichowensis*, *O. schmackeri*, *O. tianmuii* and several cryptic species (Li et al. 2015; Zhu 2016; Li et al. 2018a). Zhu (2016) considered *O. nanjiangensis* to be a synonym of *O. hejiangensis* indicating that the taxonomic status of *O. nanjiangensis* should be re-examined.

The Wuling Mountains are a priority area for biodiversity conservation in China (Ministry of Ecology and Environment of China 2015). During a survey of herpetological species diversity in Sangzhi County, Zhangjiajie City, Hunan Province of northeast Wuling Mountains, five specimens of *Odorrana* were collected in July 2019. These specimens were similar to species in the *O. schmackeri* species complex in morphology, but detailed taxonomic comparisons showed that these specimens differ from the other known species and appear to represent an independent evolutionary lineage of the genus *Odorrana*. The results indicate that these specimens represent a new species and that this species belongs to the *O. schmackeri* species complex.

## Materials and methods

### Sampling

**Figure 1.**
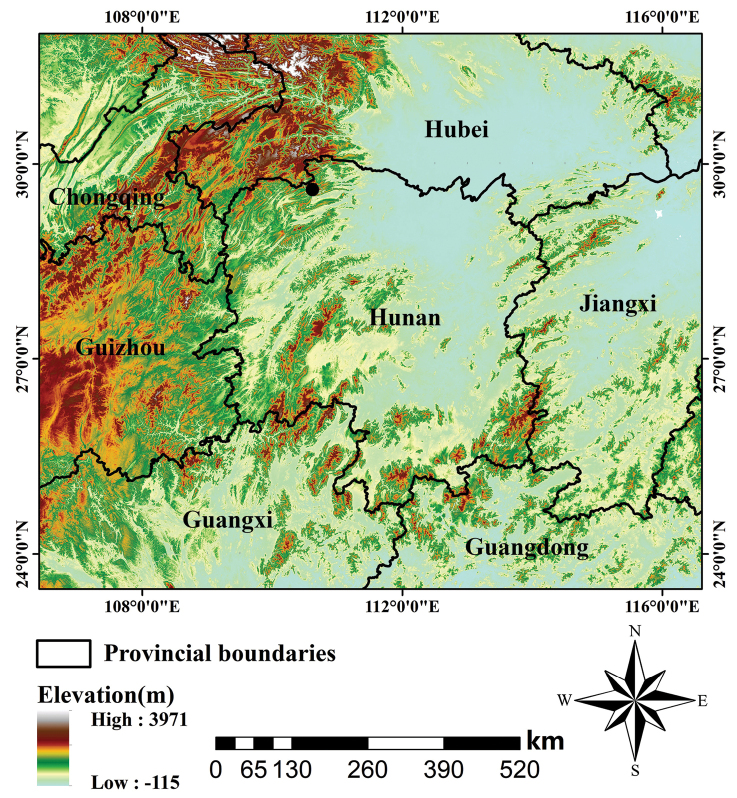
Collection location of *Odorrana sangzhiensis* sp. nov. from Sangzhi County, Hunan Province, China, indicated by a black dot.

We conducted a herpetological survey in Zhangjiajie City, Hunan Province, from 23 July to 6 August 2019. A total of five specimens (two adult males and three adult females) were collected in mountain streams on 30 July 2019, in Sangzhi County, Zhangjiajie City, Hunan Province, China (Figure 1). All of the specimens were stored in 75% ethanol (aq) for preservation and liver tissues were preserved in 95% ethanol (aq) for genetic analysis. All of the specimens were deposited at the Animal Museum of Central South University of Forestry and Technology (**CSUFT**).

### Phylogenetic analysis

Genomic DNA was extracted from 95% ethanol-preserved liver tissues using a TSINGKE (https://www.tsingke.net) DNA extraction kit. Two fragments of the mitochondrial gene 12s rRNA and 16s rRNA from five samples were amplified using the primers in Kocher et al. (1989) and Simon et al. (1994). The amplification programme followed the sequence of 98 °C for 2 min; 35 cycles of 98 °C for 10 sec, 55 °C for 10 sec, 72 °C for 10 sec; and a final extending step at 72 °C for 2 min. PCR purification and sequencing were performed by Beijing Tsingke Biological Technology Co. Ltd. For phylogenetic analyses, the available sequence data for all related species of *Odorrana* and an outgroup were downloaded from GenBank especially for the topotypes of *Odorrana* species, based on previous studies (Chen et al. 2013; Li et al. 2018a; Suppl. material 1). *Rana chensinensis* David, 1875 was used as the outgroup following Chen et al. (2013). DNA sequences were aligned by the Clustal W algorithm with default parameters (Thompson et al. 1997). Alignments were checked by eye and revised manually, if necessary. To avoid bias in alignments, GBLOCKS v. 0.91.b with default settings was used to extract regions of defined sequence conservation from the length-variable 12S and 16S gene fragments. Non-sequenced fragments were regarded as missing data. Finally, 12S and 16S gene fragments were concatenated for phylogenetic analyses of mitochondrial DNA. Sequenced data were analysed using Bayesian Inference (BI) in MrBayes 3.2.4 (Ronquist et al. 2012) and Maximum Likelihood (ML) in RaxmlGUI 1.3 (Silvestro and Michalak 2012). Prior to phylogenetic analyses, 12S and 16S genes were defined as two partitions in the concatenated data and the partitions were tested in jmodeltest v.2.1.2 with Akaike and Bayesian information criteria, all resulting in the best-fitting nucleotide substitution models of GTR+I+G. Two independent runs were conducted in a BI analysis, each of which was performed for 10,000,000 generations and sampled every 1000 generations with the first 25% samples discarded as burn-in. This resulted in a potential scale reduction factor (PSRF) of < 0.005. In ML analysis, the bootstrap consensus tree inferred from 1000 replicates was utilised to represent the evolutionary history of the taxa analysed. Genetic distances between and within species were calculated in MEGA 7 using the uncorrected *p*-distance model, based on the 16S rRNA gene (Kumar et al. 2016).

### Morphological analysis

Measurements were taken by a digital caliper (0.01 mm, rounded to the nearest 0.1 mm). Character measurements followed those of Fei et al. (2009b) and Li et al. (2018a):

**SVL** snout-vent length (distance from tip of snout to posterior margin of vent);

**HDL** head length (distance from the tip of the snout to the articulation of jaw);

**HDW** maximum head width (greatest width between the left and right articulations of jaw);

**SL** snout length (distance from the tip of the snout to the anterior corner of the eye);

**ED** eye diameter (distance from the anterior corner to the posterior corner of the eye);

**IOD** interorbital distance (minimum distance between the inner edges of the upper eyelids);

**IND** internasal distance (minimum distance between the inner margins of the external nares);

**NED** nasal to eye distance (distance between the nasal margin and the anterior corner of the eye);

**TYD** maximal tympanum diameter;

**LAL** length of lower arm and hand (distance from the elbow to the distal end of finger III);

**LW** lower arm width (maximum width of the lower arm);

**THL** thigh length (the distance from the vent to the knee);

**TL** tibia length (the distance from the knee to the tarsus);

**TW** maximal tibia width;

**TFL** length of foot and tarsus (distance from the tibiotarsal articulation to the distal end of toe IV);

**FL** foot length (distance from tarsus to the tip of toe IV);

**FDW** width of finger disc (width at the widest part of the disc of finger III).

Sexual size dimorphism was represented by the size dimorphism index (**SDI**). **SDI** was calculated as the mean of SVL of the larger sex/mean of SVL in the smaller sex – 1 (Kupfer 2009).

Comparative morphological data of four species (*Odorrana hejiangensis*, *O. huanggangensis*, *O. kweichowensis* and *O. schmackeri*) allocated to the *Odorrana schmackeri* species complex (currently containing five species) were obtained from examination of museum specimens (see Appendix 1) and from literature (Suppl. material 2) (Li et al. 2018a).

To reduce the impact of allometry, a corrected value from the ratio of each morphological measurement to SVL was calculated and was log-transformed for the following morphometric analyses. An independent sample t-test was used to test the significance of differences on morphometric characters between the new species and different species from the *Odorrana schmackeri* species complex. The sample size of male specimens was less than three, so we only analysed the morphometric data of females of the new species. Scores were considered significant at *p* < 0.05. To show the spatial distribution of different species on the morphometric characters, principal component analyses (PCA) were performed. We used one-way ANOVA to compare the morphometric character differences amongst the new species, *O. hejiangensis*, *O. huanggangensis*, *O. kweichowensis* and *O. schmackeri* and then selected the characters with significant differences amongst species for principal component analyses. All statistical analyses were conducted in R v. 3.5.1. Since the collection location of new species was about 855 km away from the distribution area of *O. tianmuii* and the distribution area of these two species was also separated by the distribution area of *O. schmackeri* (Li et al. 2018a), we did not use an independent samples t-test and PCA to compare the morphometric data between the new species and *O. tianmuii*.

We compared the morphological characters between the new species and its congeners, based on literature values from the following species: *Odorrana absita* (Stuart & Chan-ard, 2005), *O. amamiensis* (Matsui, 1994), *O. andersonii* (Boulenger, 1882), *O. anlungensis* (Hu et al., 1973), *O. aureola* (Stuart et al., 2006), *O. bacboensis* (Bain et al., 2003), *O. banaorum* (Bain et al., 2003), *O. bolavensis* (Stuart & Bain, 2005), *O. cangyuanensis* (Yang, 2008), *O. chapaensis* (Bourret, 1937), *O. chloronota* (Günther, 1876; Che et al. 2020), *O. exiliversabilis* (Fei et al., 2001b), *O. fengkaiensis* (Wang et al., 2015), *O. geminata* (Bain et al., 2009), *O. gigatympana* (Orlov et al., 2006), *O. grahami* (Boulenger, 1917), *O. graminea* (Boulenger, 1900), *O. hainanensis* (Fei et al., 2001a), *O. hejiangensis* (Deng & Yu, 1992), *O. hosii* (Boulenger, 1891), *O. huanggangensis* (Chen et al., 2010a), *O. indeprensa* (Bain & Stuart, 2005), *O. ishikawae* (Stejneger, 1901), *O. jingdongensis* (Fei et al., 2001a), *O. junlianensis* (Fei & Ye, 2001), *O. khalam* (Stuart et al., 2005), *O. kuangwuensis* (Hu et al., 1966), *O. kweichowensis* (Li et al., 2018a), *O. leporipes* (Werner, 1930), *O. lipuensis* (Mo et al., 2015), *O. livida* (Blyth, 1856), *O. lungshengensis* (Liu & Hu, 1962), *O. macrotympana* (Yang, 2008), *O. margaretae* (Liu, 1950), *O. mawphlangensis* (Pillai & Chanda, 1977), *O. monjerai* (Matsui & Jaafar, 2006), *O. morafkai* (Bain et al., 2003), *O. mutschmanni* (Pham et al., 2016), *O. nanjiangensis* (Fei et al., 2007b), *O. narina* (Stejneger, 1901), *O. nasica* (Boulenger, 1903), *O. nasuta* (Fei et al., 2001b), *O. orba* (Stuart & Bain, 2005), *O. rotodora* (Yang, 2008), *O. schmackeri* (Boettger, 1892), *O. sinica* (Bain et al., 2003), *O. splendida* (Kuramoto et al., 2011), *O. supranarina* (Matsui, 1994), *O. swinhoana* (Boulenger, 1903), *O. tianmuii* (Chen et al. 2010b), *O. tiannanensis* (Yang & Li, 1980), *O. tormota* (Wu, 1977), *O. trankieni* (Orlov et al., 2003), *O. utsunomiyaorum* (Matsui, 1994), *O. versabilis* (Liu & Hu, 1962), *O. wuchuanensis* (Wu et al., 1983), *O. yentuensis* (Tran et al., 2008) and *O. yizhangensis* (Fei et al., 2007a).

## Results

### Phylogenetic analyses

**Figure 2.**
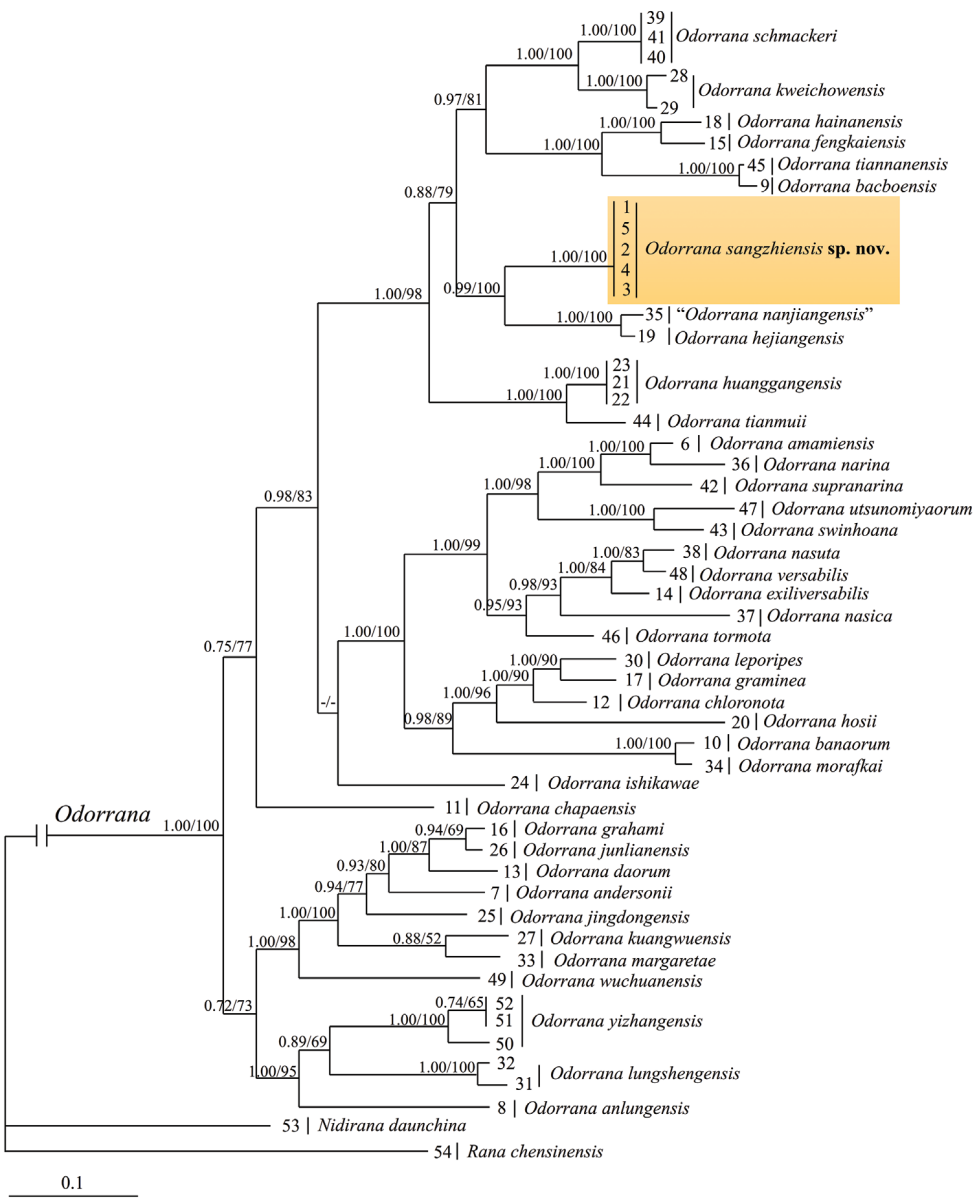
Bayesian Inference tree based on the partial DNA sequences of the mitochondrial 12S and 16S genes. Numbers before slashes indicate Bayesian posterior probabilities and numbers after slashes are bootstrap support for Maximum Likelihood (1000 replicates) analyses. The symbol “-” indicates a value below 50%.

The ML and BI phylogenetic trees were constructed, based on DNA sequences of mitochondrial 12S and 16S genes with a total length of 1,960 bp (Figure 2). The validity of the unnamed *Odorrana* specimens was affirmed by molecular phylogenetic analysis. All five samples of the new taxon occurring in Sangzhi County were densely clustered into a strongly-supported monophyletic group. The phylogenetic tree suggested that the new taxon is sister to *O. hejiangensis* and *O. nanjiangensis* and belongs to the *O. schmackeri* species complex. The uncorrected *p*-distances between the new taxon and all other species of the complex are 2.8%–9.8%, which were larger than the interspecific genetic distance between, for example, *O. fengkaiensis* and *O. hainanensis*, *O. huanggangensis* and *O. tianmuii* and *O. nasuta* and *O. versabilis* (Suppl. material 3).

### Taxonomic account

#### Odorrana sangzhiensis

Taxon classificationAnimaliaAnuraRanidae

Zhang, Li, Hu & Yang sp. nov.

B0303ABF-669B-5D64-BA36-8FFFDED3800C

http://zoobank.org/3819587B-C4B1-46F8-9FB8-7B25D5FA9A71

##### Holotype.

**Figure 3.**
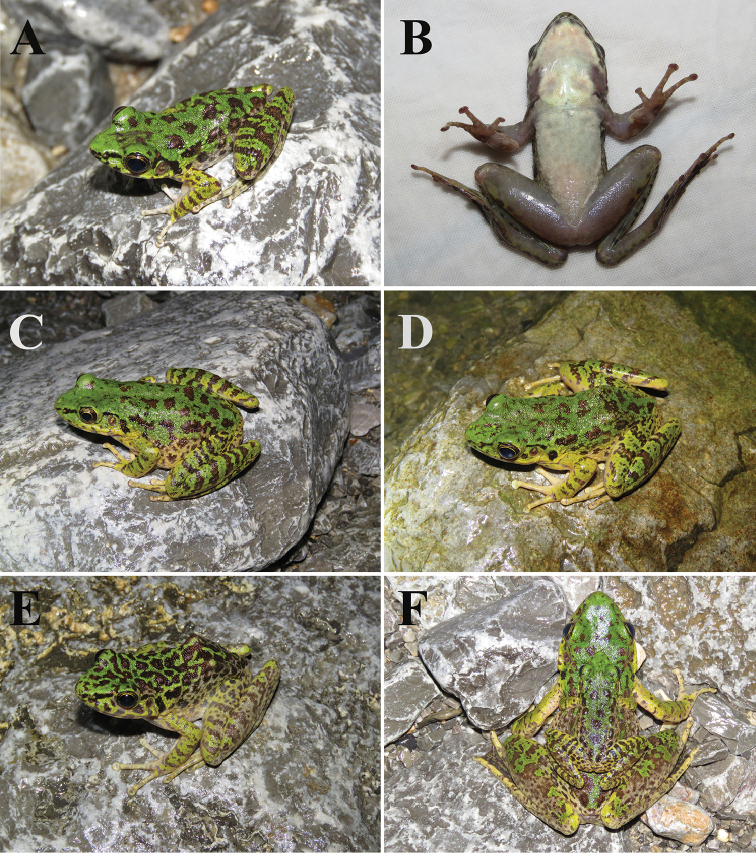
Morphology and colour pattern of living *Odorrana sangzhiensis* sp. nov. **A** dorsolateral view of the holotype CSUFT 4308220051 **B** ventral view of the holotype CSUFT 4308220051 **C** dorsolateral view of a gravid female (CSUFT 4308220047) **D** dorsolateral view of adult female **E** variation in morphology and colour pattern of an adult female (CSUFT 4308220048) **F** reproductive behaviour of *Odorrana sangzhiensis* sp. nov.

CSUFT 4308220051, adult male collected by Bing Zhang on 30 July 2019, in Sangzhi County, Zhangjiajie City, Hunan Province, China (29°38'54.66"N, 110°34'44.62"E; 540 m a.s.l.; Figure 3A, B).

##### Paratypes.

Three adult females, CSUFT 4308220046, CSUFT 4308220047 (Figure 3C) and CSUFT 4308220048 (Figure 3E); one adult male, CSUFT 4308220049; the same collection information as the holotype.

##### Diagnosis.

*Odorrana sangzhiensis* sp. nov. can be distinguished from all of its congeners by a combination of following characters: (1) medium size, SDI = 1.03 (SVL 83.3–92.7 mm in females and 42.1–45.1 mm in males); (2) head length greater than head width; (3) dorsolateral folds absent; (4) a small white or beige dot between eyes; (5) brown supratympanic fold slightly distinct; (6) the tympanum diameter in females 1.53 times as long as the width of the disc of finger III; in males 2.3 times; (7) green dorsal skin with dense granules and sparse, irregular brown spots; the males with several large warts on dorsum; (8) flanks with larger tubercles, 4–7 large tubercles usually arranged in a dorsolateral row of the males; (9) two metacarpal tubercles; (10) discs of all digits with circum-marginal grooves; (11) relative finger lengths: I ≤ II < IV < III; (12) feet fully webbed; (13) tibiotarsal articulation beyond the tip of the snout; (14) ventre smooth in females; pale spinules present on throat and chest of adult males; (15) dorsal digits light yellow or beige with light brown spots, dorsal limbs green or yellow green with brown transverse bands; (16) having paired external vocal sacs located at corners of the throat, light yellow nuptial pad on finger I in males.

##### Description of holotype.

Adult male, SVL 42.1 mm; top of head flat; head length greater than maximum head width (HDL: HDW = 1.36); snout obtusely rounded in dorsal view (SL/HDL = 0.39), rounded in profile, projecting beyond lower jaw; large and protruding eyes (ED/SL = 0.79), deeply concave loreal region; IND = 5.3 mm, larger than IOD = 3.9 mm; nasal margin slightly closer to the tip of snout; tympanum circular, large and distinct, surrounded by many granules, tympanum diameter 0.65 times the eye diameter; vomerine teeth developed into mass on two oblique ridges on the inside of two internal nostrils; tongue deeply notched posteriorly.

Forelimbs sturdy (LW/LAL = 0.21); length of lower arm and hand just over half the body length (LAL/SVL > 0.50); relative finger lengths: I ≤ II < IV < III; FDW/TYD = 0.44; finger webbing absent, tips of fingers moderately expanded, presenting circular discs with slightly narrow top and circum-marginal grooves; subarticular tubercles prominent; supernumerary tubercle at the base of each finger smaller than subarticular tubercles; two oblong metacarpal tubercles; buff nuptial pad on the first finger.

Hind limbs relatively long, the heels overlapping obviously, tibiotarsal articulation beyond the tip of the snout; tibia length (TL) 0.54 times SVL; foot length (FL) 0.56 times SVL; toes, slender, relative toe lengths I < II < III < V < IV; tibia length slightly shorter than foot length; subarticular tubercles prominent; toes discs same as fingers; obvious horizontal groove in dorsal view of discs; feet fully webbed to discs; lateral fringes on free edges of toes I and V not obvious, metatarsal fold absent; inner metatarsal tubercle present, elliptical; outer metatarsal tubercle absent; inner tarsal fold absent.

Skin slightly rough with dense granules, several large warts on dorsum and flanks; dorsolateral folds absent; two glands behind the back edge of the lips; a small beige dot between anterior corners of the eyes; pale spinules present on throat and chest.

##### Colouration in life.

In life, the yellow-green dorsum of head and body with scattered irregular dark brown spots; no light edges around spots; small area of dark brown spots on the dorsum of head; large dark black spots in the centre on dorsum, continuing on to light yellow flank with several brown spots; supratympanic fold slightly distinct, brown; upper and lower lip with vertical brown bars; arms green-yellow with brown transverse bands, thighs and tibias with four brown bands; different widths of brown striations on green-yellow limbs, different distances between striations (Figure 3).

##### Colouration in alcohol.

**Figure 4.**
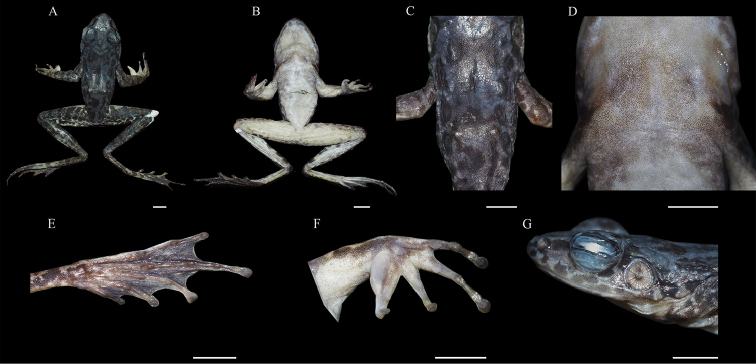
The holotype CSUFT 4308220051 of *Odorrana sangzhiensis* sp. nov. in preservative **A** dorsal view of the holotype **B** ventral view of the holotype **C** view of dorsum **D** view of chest **E** ventral view of the right foot **F** ventral view of the right hand **G** lateral view of the head. Scale bars: 0.5 cm.

On dorsum, colour fades to grey-blue with dark grey spots, brown bars on upper and lower lips change to dark grey; ventre variable from yellowish to creamy-white; beige dot between anterior corners of the eyes fades to white; underside of limbs varies from brown to beige (Figure 4).

##### Variation.

Measurements of the five specimens are shown in Table 1. The specimens show obvious differences between females and males in each character measurement. The snout-vent length of females is approximately twice as long as the males (SVL mean 88.6 mm, range 83.3–92.7 mm in females; SVL mean 43.6 mm, range 42.1–45.1 mm in males). In the ratio of each character to SVL, HDL, HDW, ED, IOD, TYD and LW, these are all significantly smaller in females than in males. Some females have dense brown spots on the dorsum and most of the yellow green on their limbs is replaced by yellow white (Figure 3E).

**Table 1.** Measurements (mm) of the morphometric characters for the holotype and paratypes of *Odorrana sangzhiensis* sp. nov. (H = holotype, P = paratype, for other abbreviation, see Materials and methods).

Specimen ID	CSUFT 4308220051	CSUFT 4308220049		CSUFT 4308220046	CSUFT 4308220047	CSUFT 4308220048		
Sex	Male	Male	Mean (n=2)	Female	Female	Female	Min–Max (n=3)	Mean±SD (n=3)
Type status	H	P		P	P	P		
SVL	42.1	45.1	43.6	89.7	92.7	83.3	83.3–92.7	88.6 ± 4.8
HDL	16.5	15.2	15.9	25.8	25.8	23.6	23.6–25.8	25.1 ± 1.3
HDW	12.1	12.6	12.4	21.4	22.2	21.9	21.4–22.2	21.8 ±0.4
SL	6.5	5.8	6.2	12.3	11.0	11.8	11.0–12.3	11.7 ±0.7
ED	5.2	5.6	5.4	8.5	8.4	8.1	8.1–8.5	8.3 ± 0.2
IOD	3.9	4.6	4.3	6.5	7.4	6.5	6.5–7.4	6.8 ± 0.5
IND	5.3	4.8	5.1	8.7	8.7	8.9	8.7–8.9	8.8 ± 0.1
NED	3.4	3.5	3.5	5.8	6.5	6.8	5.8–6.8	6.4 ± 0.5
TYD	3.4	3.3	3.4	4.6	4.9	4.3	4.3–4.9	4.6 ± 0.3
LAL	21.1	22.8	22.0	42.3	41.0	41.4	41.0–42.3	41.6 ± 0.7
LW	4.5	4.7	4.6	8.4	8.9	7.1	7.1–8.9	8.1 ± 0.9
THL	21.6	25.9	23.8	46.9	50.2	47.6	46.9–50.2	48.2 ± 1.7
TL	22.8	23.6	23.2	46.3	47.6	51.0	46.3–51.0	48.3 ± 2.4
TW	5.0	5.6	5.3	12.1	12.4	12.4	12.1–12.4	12.3 ± 0.2
TFL	31.5	31.1	31.3	69.0	61.4	53.2	53.2–69.0	61.2 ± 7.9
FL	23.4	24.0	23.7	50.8	47.3	46.8	46.8–50.8	48.3 ± 2.2
FDW	1.5	1.4	1.5	3.0	3.3	2.7	2.7–3.3	3.0 ± 0.3

##### Comparisons.

*Odorrana sangzhiensis* sp. nov. can be distinguished from *O. absita*, *O. amamiensis*, *O. banaorum*, *O. bolavensis*, *O. exiliversabilis*, *O. gigatympana*, *O. graminea*, *O. hosii*, *O. indeprensa*, *O. khalam*, *O. leporipes*, *O. livida*, *O. monjerai*, *O. narina*, *O. nasica*, *O. nasuta*, *O. orba*, *O. supranarina*, *O. tormota*, *O. trankieni*, *O. utsunomiyaorum*, *O. versabilis* and *O. yentuensis* by lacking dorsolateral folds (vs. present in other species).

*Odorrana sangzhiensis* sp. nov. differs from *O. amamiensis* (56–69 mm), *O. andersonii* (68–76 mm), *O. aureola* (63.6–67.4 mm), *O. bacboensis* (54.9 mm), *O. cangyuanensis* (62–69 mm), *O. chapaensis* (73–83 mm), *O. geminata* (71–79 mm), *O. grahami* (70–84 mm), *O. ishikawae* (88–106 mm), *O. jingdongensis* (62–82 mm), *O. junlianensis* (73–80 mm), *O. kuangwuensis* (57 mm), *O. lungshengensis* (60–67 mm), *O. macrotympana* (50.6 mm), *O. margaretae* (78–88 mm), *O. mawphlangensis* (80 mm), *O. mutschmanni* (85.9–91.6 mm), *O. nanjiangensis* (50.0–59.6 mm), *O. splendida* (92.3–105.6 mm), *O. swinhoana* (47.7–71.5 mm), *O. trankieni* (76.1–77.0 mm) and *O. wuchuanensis* (62–77 mm) by the smaller snout-vent length in males (SVL 42.1–45.1 mm).

*Odorrana sangzhiensis* sp. nov. differs from *O. anlungensis* (II < I ≈ IV < III), *O. chloronota* (II < I < IV < III), *O. morafkai* (II < I < IV < III) and *O. rotodora* (II < I < IV < III) by the relative finger lengths I ≤ II < IV < III; from *O. sinica* (tympanum indistinct) by tympanum distinct; from *O. lipuensis* (vocal sacs absent in males) by paired external vocal sacs located at corners of the throat; from *O. lungshengensis* by tympanum diameter larger than width at the widest part of the disc of finger III (vs. tympanum diameter approximately equal to width at the widest part of the disc of finger III); from *O. yizhangensis* by the body size of females with minimum SVL 83.3 mm (vs. SVL 58.2–71.5 mm of females in the latter), the relative finger lengths I ≤ II < IV < III (vs. relative finger lengths I < II < IV < III) and tibiotarsal articulation beyond the tip of the snout (vs. only reaching the tip of the snout).

**Figure 5.**
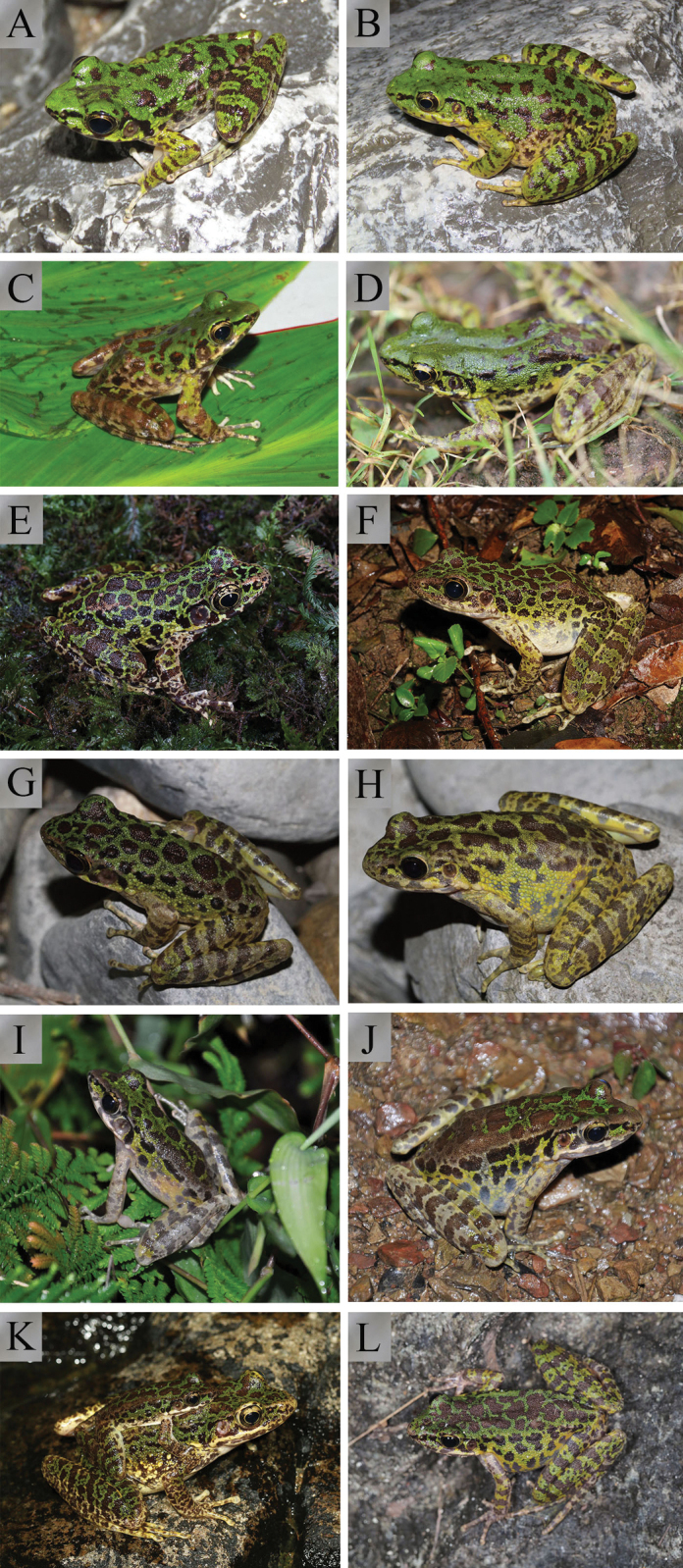
Comparisons of morphological characteristics with congeners **A***Odorrana sangzhiensis* sp. nov. (male, photo by Bing Zhang in the type locality) **B***Odorrana sangzhiensis* sp. nov. (female, photo by Bing Zhang in the type locality) **C***Odorrana hejiangensis* (male, photo by Shize Li in Chishui City, Guizhou Province, China) **D***Odorrana hejiangensis* (female, photo by Bo Cai in the type locality) **E***Odorrana huanggangensis* (male, photo by Shengchao Shi in the type locality) **F***Odorrana huanggangensis* (female, photo by Shengchao Shi in the type locality) **G***Odorrana kweichowensis* (male, photo by Shize Li in the type locality) **H***Odorrana kweichowensis* (female, photo by Shize Li in the type locality) **I***Odorrana schmackeri* (male, photo by Liang Qiao in Wushan County, Chongqing City, China) **J***Odorrana schmackeri* (female, photo by Liang Qiao in Wushan County, Chongqing City, China) **K***Odorrana tianmuii* (male, photo by Yufan Wang in the type locality) **L***Odorrana tianmuii* (female, photo by Yufan Wang in the type locality).

Within the *Odorrana schmackeri* species complex (*O. hejiangensis*, *O. huanggangensis*, *O. kweichowensis*, *O. schmackeri* and *O. tianmuii*) (Figure 5) and its relatives (*O. fengkaiensis*, *O. hainanensis* and *O. tiannanensis*), *Odorrana sangzhiensis* sp. nov. differs from *O. hejiangensis* by the body size of males with maximum SVL 45.1 mm (vs. SVL 50.7–57.8 mm of males in the latter), the larger size dimorphism index (SDI = 1.03 vs. SDI = 0.49), the head longer than wider (vs. head longer or almost equally wide), two metacarpal tubercles (vs. three metacarpal tubercles), discs of all digits with circum-marginal grooves (vs. without circum-marginal grooves on finger I in the latter), tibiotarsal articulation beyond the tip of the snout (vs. reaching to the level between eye to nostril) and the relative finger lengths I ≤ II < IV < III (vs. relative finger lengths II < I < IV < III) (Table 2); from *O. huanggangensis* by the head longer than wide (vs. head longer to almost equally wide), two metacarpal tubercles (vs. three metacarpal tubercles), tibiotarsal articulation beyond the tip of the snout (vs. just reaching the nostril) and the relative toe lengths I < II < III < V < IV (vs. relative toe lengths I < II < III ≤ V < IV) (Table 2); from *O. kweichowensis* by the body size of females with minimum SVL 83.3 mm (vs. SVL 67.9–78.7 mm of females in the latter), tibiotarsal articulation beyond the tip of the snout (vs. reaching the level between the anterior corner of the eye and the nostril) and the relative finger lengths I ≤ II < IV < III (vs. relative finger lengths II < I < IV < III) (Tables 2, 3); from *O. schmackeri* by temporal fold non-prominent (vs. temporal fold prominent), the head longer than wide (vs. head longer almost equally wide), two metacarpal tubercles (vs. outer metacarpal tubercle being indistinct), tibiotarsal articulation beyond the tip of the snout (vs. reaching to the level between eye to nostril), the relative finger lengths I ≤ II < IV < III (vs. relative finger lengths II < I ≈ IV < III) and the relative toe lengths I < II < III < V < IV (vs. relative toe lengths I < II < III ≈ V < IV) (Table 2); from *O. tianmuii* by two metacarpal tubercles (vs. three metacarpal tubercles), tibiotarsal articulation beyond the tip of the snout (vs. reaching to the level between eye to nostril) and the relative toe lengths I < II < III < V < IV (vs. relative toe lengths I < II < III ≤ V < IV) (Table 2); from *O. fengkaiensis* by two metacarpal tubercles (vs. three metacarpal tubercles) and the relative finger lengths I ≤ II < IV < III (vs. relative finger lengths II < I < IV < III) (Table 2); from *O. hainanensis* by two metacarpal tubercles (vs. three metacarpal tubercles), paired external vocal sacs located at corners of the throat (vs. paired internal vocal sacs in males), tibiotarsal articulation beyond the tip of the snout (vs. reaching the anterior corner of the eye or the tip of snout) and the relative finger lengths I ≤ II < IV < III (vs. relative finger lengths II < IV ≈ I < III) (Table 2); from *O. tiannanensis* by the body size of males with maximum SVL 45.1 mm (vs. SVL 52.5–53.5 mm of males in the latter), tibiotarsal articulation beyond the tip of the snout (vs. far beyond the snout) and the relative finger lengths I ≤ II < IV < III (vs. relative finger lengths II < I < IV < III) (Tables 2, 3).

**Table 2.** Diagnostic characters separating the new species described in this study and its relatives.

Species	SVL		Head length^1^	Metacarpal tubercles^2^	Circum-marginal grooves^3^	Vocal sacs^4^	Tibiotarsal articulation^5^	Relative finger lengths^6^	Relative toe lengths^7^
	males (n)	females (n)							
*Odorrana sangzhiensis* sp. nov.	42.1–45.1 (2)	83.3–92.7 (3)	++	+	+	+	a	a	a
*Odorrana hejiangensis*	50.7–57.8 (8)	73.3–84.9 (5)	+	++	–	+	b	b	a
*Odorrana huanggangensis*	38.0–46.7 (7)	76.9–88.6 (6)	–	++	+	+	c	a	b
*Odorrana kweichowensis*	36.2–43.3 (7)	67.9–78.7 (6)	++	+	+	+	b	b	a
*Odorrana schmackeri*	37.9–43.4 (7)	72.1–80.4 (4)	–	–	+	+	b	c	c
*Odorrana tianmuii*	39.4–45.9 (23)	68.1–81.9 (8)	++	++	+	+	b	a	b
*Odorrana fengkaiensis*	37.4–51.8 (11)	77.3–111.9 (31)	++	++	+	/	a	b	a
*Odorrana hainanensis*	49.1–61.9 (5)	75.4–122.5 (8)	++	++	+	–	d	d	a
*Odorrana tiannanensis*	52.5–53.5 (2)	90.5–107.6 (4)	++	/	+	+	e	b	/

^1^ head longer than wider (++), head longer or almost equal wide (+), head longer almost equal wide (–); ^2^ three metacarpal tubercles (++), two metacarpal tubercles (+), outer metacarpal tubercle being indistinct (–); ^3^ present (+), without circum-marginal grooves on finger I (–), outer metacarpal tubercle being indistinct (–); ^4^ paired external vocal sacs (+), paired internal vocal sacs (–) in males; ^5^ beyond the tip of the snout (a), reaching to the level between eye to nostril (b), reaching the nostril (c), reaching the anterior corner of the eye or the tip of snout (d), far beyond the snout (e); ^6^ I ≤ II < IV < III (a), II < I < IV < III (b), II < I ≈ IV < III (c), and II < IV ≈ I < III (d); ^7^ I < II < III < V < IV (a), I < II < III ≤ V < IV (b), and I < II < III ≈ V < IV (c).

**Table 3.** Morphometric comparisons between *Odorrana sangzhiensis* sp. nov. and its relatives (Males). See abbreviations for characters in the Materials and methods section.

Characters	*Odorrana sangzhiensis* sp. nov.	*Odorrana hejiangensis*	*Odorrana huanggangensis*	*Odorrana kweichowensis*	*Odorrana schmackeri*
	Males (n = 2)	Males (n = 8)	Males (n = 7)	Males (n = 7)	Males (n = 7)
SVL	42.1–45.1	50.7–57.8 (54.3 ± 2.4)	38.0–46.7 (42.2 ± 2.8)	36.2–43.3 (40.9 ± 2.6)	37.9–43.4 (41.6 ± 2.0)
HDL	15.2–16.5	19.2–21.3 (20.3 ± 0.8)	14.2–18.0 (16.0 ± 1.2)	14.1–17.3 (15.9 ± 1.3)	15.4–18.0 (17.2 ± 0.9)
HDW	12.1–12.6	17.9–21.3 (19.1 ± 1.1)	12.7–16.0 (14.9 ± 1.1)	12.5–15.7 (14.4 ± 1.1)	13.9–15.8 (15.0 ± 0.7)
SL	5.8–6.5	8.4–10.1 (9.0 ± 0.6)	5.9–7.4 (6.7 ± 0.6)	5.5–7.0 (6.5 ± 0.5)	6.1–7.3 (6.9 ± 0.4)
ED	5.2–5.6	6.0–7.3 (6.6 ± 0.4)	4.4–6.5 (5.7 ± 0.7)	4.8–5.9 (5.5 ± 0.5)	3.6–6.9 (5.8 ± 1.1)
IOD	3.9–4.6	4.2–5.3 (4.6 ± 0.3)	3.0–3.9 (3.3 ± 0.4)	2.6–3.5 (3.1 ± 0.3)	3.2–4.1 (3.6 ± 0.3)
IND	4.8–5.3	5.0–6.7 (5.7 ± 0.6)	4.1–5.3 (4.6 ± 0.4)	4.5–5.4 (4.9 ± 0.3)	4.5–5.1 (4.8 ± 0.2)
NED	3.4–3.5	4.2–6.0 (4.9 ± 0.6)	3.0–4.2 (3.6 ± 0.4)	3.1–4.0 (3.6 ± 0.4)	3.3–4.2 (3.8 ± 0.3)
TYD	3.3–3.4	4.3–5.5 (4.6 ± 0.4)	3.3–4.4 (4.0 ± 0.5)	2.9–4.2 (3.8 ± 0.4)	3.3–4.6 (4.1 ± 0.4)
LAL	21.1–22.8	24.8–26.8 (26.0 ± 0.7)	18.5–21.9 (20.3 ± 1.4)	20.2–24.3 (21.4 ± 1.4)	19.6–22.2 (20.4 ± 0.9)
LW	4.5–4.7	5.0–6.3 (5.6 ± 0.5)	3.1–5.0 (4.2 ± 0.8)	3.7–4.4 (4.0 ± 0.2)	3.8–5.0 (4.3 ± 0.4)
THL	21.6–25.9	25.6–30.1 (27.8 ± 1.6)	19.6–24.0 (22.2 ± 1.4)	19.6–25.2 (23.0 ± 2.2)	20.8–24.6 (22.4 ± 1.1)
TL	22.8–23.6	30.4–33.9 (31.8 ± 1.0)	22.6–27.0 (24.7 ± 1.6)	22.7–28.8 (25.2 ± 2.0)	23.4–25.0 (24.4 ± 0.6)
TW	5.0–5.6	6.4–7.4 (6.8 ± 0.4)	4.7–5.9 (5.3 ± 0.4)	4.4–5.6 (5.1 ± 0.5)	5.0–6.0 (5.6 ± 0.3)
TFL	31.1–31.5	41.2–43.2 (42.5 ± 0.7)	29.3–35.9 (32.3 ± 2.3)	30.1–38.7 (34.7 ± 2.6)	30.6–36.4 (33.2 ± 1.8)
FL	23.4–24.0	28.0–30.8 (29.3 ± 1.0)	20.4–25.9 (23.3 ± 2.0)	21.1–26.8 (24.1 ± 1.8)	22.7–24.6 (23.6 ± 0.6)
FDW	1.4–1.5	1.4–2.1 (1.7 ± 0.2)	1.2–2.1 (1.6 ± 0.3)	1.2–1.9 (1.5 ± 0.2)	1.4–2.0 (1.6 ± 0.2)

**Figure 6.**
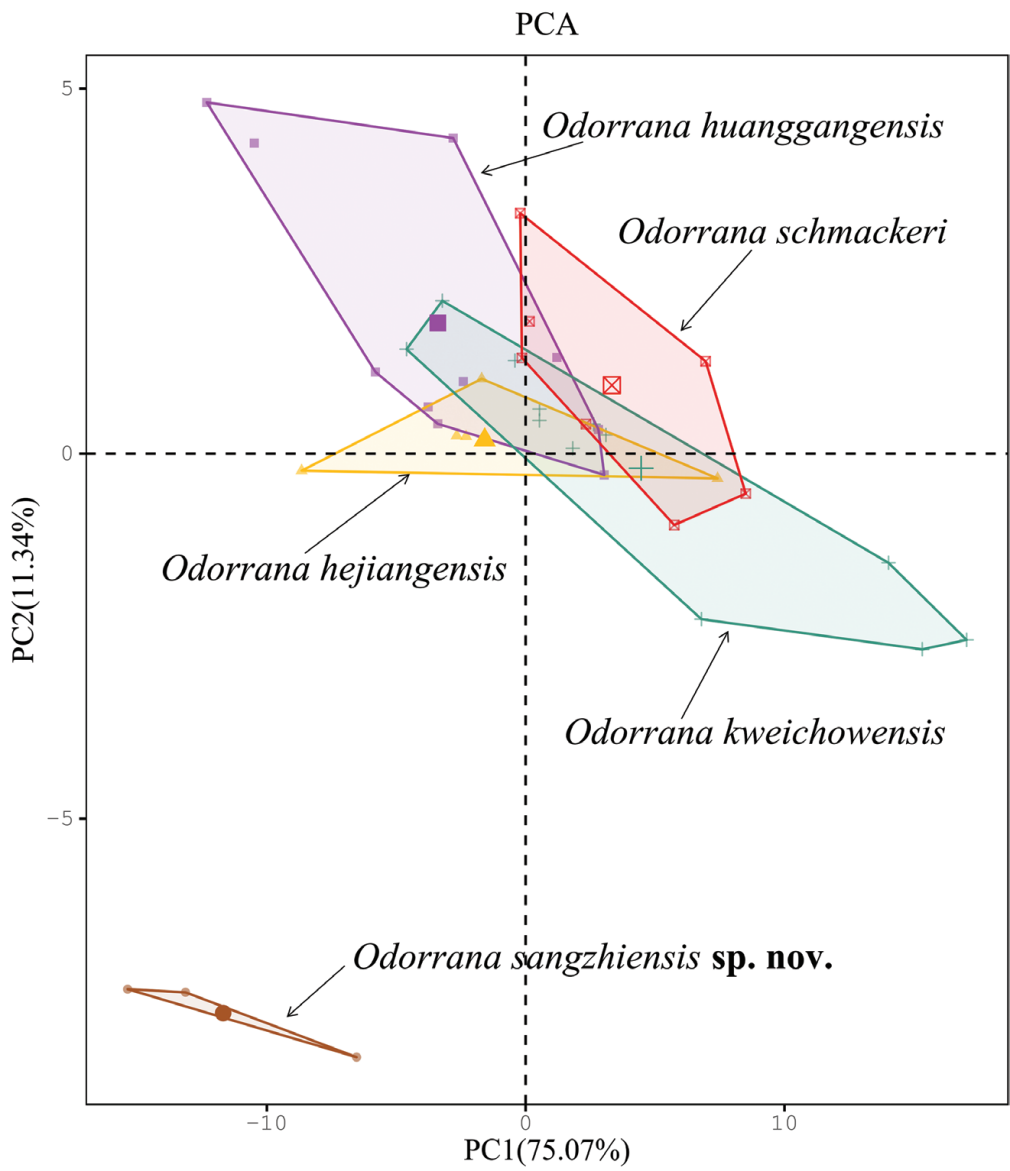
Plots of principal component analyses of *Odorrana sangzhiensis* sp. nov., *O. hejiangensis*, *O. huanggangensis*, *O. kweichowensis* and *O. schmackeri*. Different species were denoted as different colours and shapes.

*Odorrana sangzhiensis* sp. nov. differs from *O. hejiangensis* by having significantly lower ratios of HDL, HDW, SL, TYD and TFL to SVL in females (all *p*-values < 0.05; Table 4); from *O. huanggangensis* by having significantly lower ratios of HDL, HDW, SL, ED, TFL and FDW to SVL in females (all *p*-values < 0.05; Table 4); from *O. kweichowensis* by having significantly lower ratios of HDL, HDW, SL, ED, IND, NED, TYD, LAL, TL, TFL, FL and FDW to SVL in females (all *p*-values < 0.05; Table 4); from *O. schmackeri* by having significantly lower ratios of HDL, HDW, SL, TYD and TFL to SVL in females (all *p*-values < 0.05; Table 4). In females, on the two-dimensional plots of PC1 vs. PC2, the new taxon can be almost separated from *O. hejiangensis*, *O. huanggangensis*, *O. kweichowensis* and *O. schmackeri* (Figure 6).

**Table 4.** Morphometric comparisons between *Odorrana sangzhiensis* sp. nov. and its relatives (Females). The results of independent samples t-test with *p*-values between the new species and each relative and one-way ANOVA with *P*-values amongst *Odorrana sangzhiensis* sp. nov., *O. hejiangensis*, *O. huanggangensis*, *O. kweichowensis* and *O. schmackeri*. The significant level is 0.05. See abbreviations for characters in the Materials and methods section.

Characters	*Odorrana sangzhiensis* sp. nov.	*Odorrana hejiangensis*		*Odorrana huanggangensis*		*Odorrana kweichowensis*		*Odorrana schmackeri*		*P*-value
	Females (n = 3)	Females (n = 5)	*p* -value	Females (n = 10)	*p* -value	Females (n = 12)	*p* -value	Females (n = 7)	*p*-value	
SVL	83.3–92.7 (88.6 ± 4.8)	73.3–84.9 (80.6 ± 4.3)	/	75.0–88.6 (81.7 ± 4.3)	/	64.7–81.1 (74.5 ± 5.2)	/	72.1–80.7 (77.4 ± 3.4)	/	<0.001
HDL	23.6–25.8 (25.1 ± 1.3)	26.1–29.1 (27.6 ± 1.1)	<0.001	27.5–32.8 (29.9 ± 1.6)	<0.001	25.6–30.9 (28.6 ± 1.9)	<0.001	27.1–32.3 (29.2 ± 1.8)	<0.001	0.002
HDW	21.3–22.2 (21.8 ± 0.4)	27.4–28.6 (28.0 ± 0.6)	<0.001	26.9–31.9 (28.5 ± 1.7)	<0.001	23.3–29.5 (26.4 ± 1.8)	<0.001	25.7–28.0 (26.9 ± 0.9)	<0.001	<0.001
SL	11.0–12.3 (11.7 ± 0.7)	12.5–13.9 (13.0 ± 0.6)	0.009	11.8–14.4 (13.0 ± 0.7)	<0.001	10.6–12.6 (11.6 ± 0.6)	0.001	11.2–13.5 (12.1 ± 0.9)	0.005	<0.001
ED	8.1–8.5 (8.4 ± 0.2)	8.7–9.9 (9.1 ± 0.6)	0.052	7.8–10.4 (9.1 ± 1.0)	0.044	7.9–9.9 (9.1 ± 0.6)	<0.001	7.5–9.7 (8.6 ± 0.9)	0.069	0.376
IOD	6.5–7.4 (6.8 ± 0.5)	6.3–8.2 (7.1 ± 0.7)	0.086	4.7–7.4 (6.3 ± 0.8)	0.985	5.2–6.6 (5.7 ± 0.5)	0.993	4.8–7.0 (6.2 ± 0.8)	0.557	0.005
IND	8.7–8.9 (8.8 ± 0.1)	8.0–9.3 (8.6 ± 0.5)	0.243	7.7–9.8 (8.8 ± 0.7)	0.142	7.7–9.8 (8.5 ± 0.6)	0.002	7.1–8.7 (8.1 ± 0.6)	0.417	0.231
NED	5.8–6.8 (6.4 ± 0.5)	5.8–7.3 (6.6 ± 0.6)	0.236	5.8–7.1 (6.5 ± 0.4)	0.100	5.2–7.2 (6.2 ± 0.6)	0.041	5.3–7.0 (6.2 ± 0.6)	0.204	0.534
TYD	4.3–4.9 (4.6 ± 0.3)	5.4–6.3 (5.9 ± 0.4)	0.003	3.4–5.7 (4.8 ± 0.8)	0.233	4.3–5.2 (4.7 ± 0.3)	0.003	4.5–5.7 (5.0 ± 0.4)	0.007	0.002
LAL	41.0–42.2 (41.6 ± 0.7)	34.9–40.0 (37.5 ± 2.1)	0.773	36.0–41.6 (38.7 ± 1.6)	0.732	33.4–41.4 (37.5 ± 2.6)	0.026	35.0–37.4 (36.1 ± 0.8)	0.808	0.003
LW	7.1–8.9 (8.1 ± 0.9)	6.0–7.6 (6.8 ± 0.6)	0.279	6.1–8.5 (7.2 ± 0.7)	0.337	5.2–7.8 (6.4 ± 0.8)	0.155	5.8–7.2 (6.5 ± 0.5)	0.138	0.003
THL	46.9–50.1 (48.2 ± 1.7)	40.4–47.5 (43.1 ± 2.9)	0.570	41.3–46.2 (43.3 ± 1.8)	0.241	35.8–44.9 (41.4 ± 2.9)	0.324	37.7–42.8 (40.6 ± 1.9)	0.133	0.001
TL	46.2–51.0 (48.3 ± 2.5)	46.6–50.7 (48.3 ± 2.1)	0.128	41.8–53.1 (48.7 ± 3.1)	0.054	41.0–49.8 (46.3 ± 2.7)	0.001	43.0–46.5 (45.3 ± 1.2)	0.122	0.054
TW	12.1–12.4 (12.3 ± 0.2)	9.6–11.0 (10.2 ± 0.5)	0.105	10.0–13.0 (11.3 ± 1.0)	0.983	8.3–11.2 (9.9 ± 1.1)	0.256	9.6–11.2 (10.2 ± 0.6)	0.237	0.001
TFL	53.2–69.0 (61.2 ± 7.9)	58.8–68.8 (64.0 ± 4.5)	0.048	61.3–71.4 (66.2 ± 3.0)	0.001	55.9–70.0 (62.5 ± 4.3)	<0.001	60.3–63.5 (61.5 ± 1.0)	0.009	0.103
FL	46.8–50.8 (48.3 ± 2.2)	40.8–48.5 (44.9 ± 2.9)	0.537	42.6–48.3 (45.5 ± 1.8)	0.498	37.9–48.1 (44.1 ± 3.3)	0.014	40.2–43.2 (41.9 ± 1.2)	0.875	0.008
FDW	2.7–3.3 (3.0 ± 0.3)	2.4–3.3 (2.8 ± 0.3)	0.841	2.7–3.5 (3.1 ± 0.2)	0.014	2.4–3.5 (2.9 ± 0.3)	0.009	2.1–3.2 (2.6 ± 0.3)	0.927	0.039

##### Etymology.

The scientific name “*sangzhiensis*” is derived from its type locality Sangzhi County in Hunan Province. As common names, we suggest Sangzhi Odorous Frog (English) and Sang Zhi Chou Wa (Chinese).

##### Distribution and natural history.

**Figure 7.**
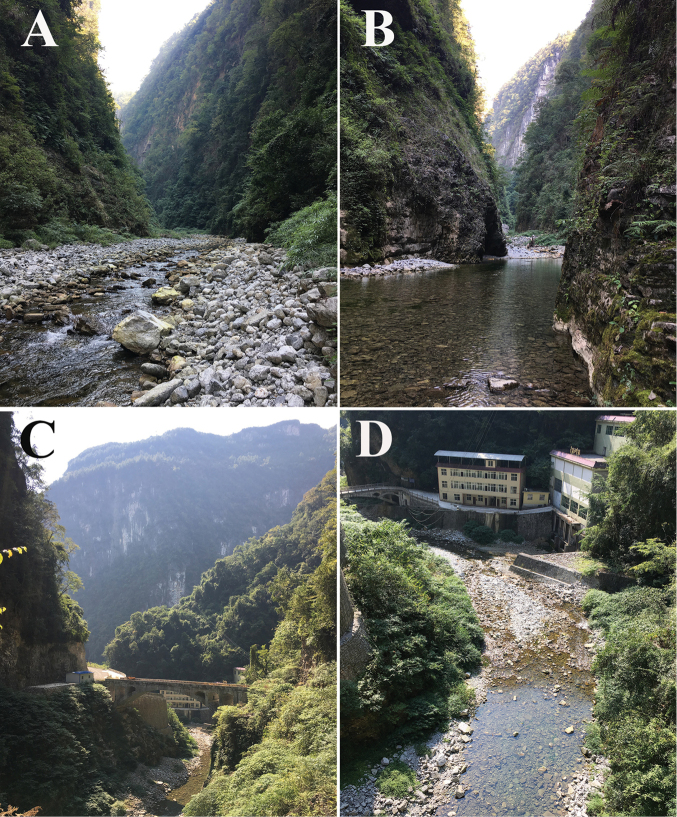
Habitat of *Odorrana sangzhiensis* sp. nov. in the type locality **A, B** canyon stream habitats in the type locality **C, D** habitats in the type locality threatened by the hydropower station.

The new species is currently known only from its type locality, Sangzhi County, Zhangjiajie, Hunan Province, China (Figure 1). It is found in canyon streams and perches on the rocks beside the streams (Figure 7A, B). They usually hide under the rocks during the day and are active at night. The vegetation on both sides of the stream is luxuriant and comprises a mixed forest of evergreen and deciduous flora. In their habitat, the dominant trees are *Ulmus changii* Cheng, 1936, *Castanopsis carlesii* Hayata, 1917 and *Sloanea hemsleyana* Rehder & Wilson, 1916 and the dominant shrubs are *Boehmeria penduliflora* Weddell & Long, 1982 and *Distylium myricoides* Hemsl, 1907. The dominant herbs are *Pilea sinofasciata* Chen, 1982, *Strobilanthes dimorphotricha* Hance, 1883 and *Miscanthus floridulus* Labillardière, 1824. One sympatric amphibian species, *Amolops ricketti* Boulenger, 1899, was found. In the field investigation, the tadpoles and eggs of this new species were not found. However, we observed reproductive behaviour (Figure 3F). In addition, one female (CSUFT 4308220047) collected on 30 July 2019, contained mature eggs (Figure 3C). The eggs in preservative showed yellowish-white. Thus, the breeding period of this species may be in July and August.

## Discussion

Odorous frog species are widely distributed and have many morphological characteristics of derivation or specialisation. Different species of *Odorrana* are distributed in the same region or in different niches within the same area, which makes species identification difficult and may also cause some cryptic species to be overlooked (Fei et al. 2009a; Li et al. 2015; Frost 2020). DNA sequence comparison and the genetic distance threshold is a highly effective method to identify amphibian species and eliminate misidentifications (Xiong et al. 2015). A cryptic species, *Odorrana kweichowensis*, has recently been described from Guizhou Province using morphological and molecular data and has morphological characteristics similar to *O. schmackeri* (Li et al. 2018a). The discovery of *Odorrana sangzhiensis* sp. nov. adds another species to the *O. schmackeri* species complex, indicating that the species diversity of *Odorrana* may be under-estimated.

The Yunnan-Guizhou Plateau is thought to be the centre of *Odorrana* origin (Ye & Fei 2001). The uplift of the Qinghai-Tibet Plateau altered the water systems and affected the geographic pattern and genetic structure of *Odorrana* species (Li et al. 2015). To clarify the species composition and distribution range of the *Odorrana schmackeri* species complex, Zhu (2016) analysed the genetic differentiation of the complex by sampling based on the partial mitochondrial 12S and 16S r RNA genes (1021 individuals from 85 populations in 15 Chinese Provinces were studied). The results showed that the *Odorrana schmackeri* species complex included four described species, i.e. *O. schmackeri*, *O. hejiangensis*, *O. huanggangensis*, *O. tianmuii* and two cyptic species. *O. hejiangensis* is mainly distributed in the northeast, northern, eastern and southeast areas of the Sichuan basin and extends to the east of Daba Mountains along the Wu Mountains and reaches the southern Funiu Mountains of Henan Province along the Wu Mountains and eastern Daba Mountains. *O. nanjiangensis* from Ningqiang Shanxi, Wenxian Shanxi and Nanjiang Sichuan Provinces was revised as *O. hejiangensis*. The distribution area of *Odorrana sangzhiensis* sp. nov. is located in the north-eastern Wuling Mountains in the east portion of the distribution area of *O. hejiangensis*. The nearest distance between the distribution area of *Odorrana sangzhiensis* sp. nov. and that of *O. hejiangensis* is about 140 km. It will be necessary to determine if the two species are sympatric or whether their reproduction period is staggered in this area. In the field investigation, we found that the habitat of this species was seriously threatened by a hydropower station (Figure 7C, D). Further investigation in this area should be conducted to determine the population status and distributional range of this species and locate additional cryptic species of *Odorrana*.

## Supplementary Material

XML Treatment for Odorrana sangzhiensis

## Appendix 1

**Specimens of comparative species examined**

*Odorrana hejiangensis* (n = 13): China: Sichuan Province: Luzhou City: Hejiang County: Zihuai Township: Shunyangxi Village (type locality): SYS a004936-38; China: Guizhou Province: Zunyi City: Chishui City: Chishui National Nature Reserve: SYS a004944, SYS a004946; China: Sichuan Province: Luzhou City: Hejiang County: Zihuai Township: Shuiliandonggou: CIB103570-74; China: Sichuan Province: Luzhou City: Hejiang County: Tiantangba Township: Yueliangyan: CIB93488-89; China: Sichuan Province: Luzhou City: Hejiang County: Renlaoqian: CIB105957.

*Odorrana huanggangensis* (n = 4): China: Guangxi Province: Guilin City: Guanyang County: Qianjiadong Nature Reserve: SYS a005098; China: Guangdong Province: Maoming City: Gaozhou City: Xianrendong Scenic Spot: SYS a005246-48.

*Odorrana schmackeri* (n = 3): China: Hubei Province: Yichang City: Changyang County: Gaojiayan: Lancaogu (type locality): SYS a005479, SYS a005481, SYS a005484.

*Odorrana fengkaiensis* (n = 3): China: Guangdong Province: Zhaoqing City: Fengkai County: Heishiding Nature Reserve (type locality): SYS a000176, SYS a002263, SYS a002265.

## Supplementary materials

Supplementary material 1 Table S1. Samples and GenBank accession numbers for molecular analyses used in this study Data type phylogenetic data https://binary.pensoft.net/file/520389This dataset is made available under the Open Database License (http://opendatacommons.org/licenses/odbl/1.0/). The Open Database License (ODbL) is a license agreement intended to allow users to freely share, modify, and use this Dataset while maintaining this same freedom for others, provided that the original source and author(s) are credited.Bing Zhang, Yuan Li, Ke Hu, Pipeng Li, Zhirong Gu, Nengwen Xiao, Daode Yang

Supplementary material 2 Table S2. The morphometric data of the *Odorrana sangzhiensis* sp. nov. and its four relatives Data type morphological data Explanation note See abbreviations for characters in the Materials and methods section. https://binary.pensoft.net/file/520390This dataset is made available under the Open Database License (http://opendatacommons.org/licenses/odbl/1.0/). The Open Database License (ODbL) is a license agreement intended to allow users to freely share, modify, and use this Dataset while maintaining this same freedom for others, provided that the original source and author(s) are credited.Bing Zhang, Yuan Li, Ke Hu, Pipeng Li, Zhirong Gu, Nengwen Xiao, Daode Yang

Supplementary material 3 Table S3. Uncorrected *p*-distances between *Odorrana* species based on mitochondrial 16S rRNA genes Data type phylogenetic data https://binary.pensoft.net/file/520391This dataset is made available under the Open Database License (http://opendatacommons.org/licenses/odbl/1.0/). The Open Database License (ODbL) is a license agreement intended to allow users to freely share, modify, and use this Dataset while maintaining this same freedom for others, provided that the original source and author(s) are credited.Bing Zhang, Yuan Li, Ke Hu, Pipeng Li, Zhirong Gu, Nengwen Xiao, Daode Yang
